# Mig‐6 could inhibit cell proliferation and induce apoptosis in esophageal squamous cell carcinoma

**DOI:** 10.1111/1759-7714.14223

**Published:** 2021-11-30

**Authors:** Yuantao Cui, Ying Kang, Peng Zhang, Yuanguo Wang, Zhaoyu Yang, Chao Lu, Peng Zhang

**Affiliations:** ^1^ Department of Cardiothoracic Surgery Tianjin Medical University General Hospital, Tianjin Medical University Tianjin China

**Keywords:** apoptosis, ESCC, Mig‐6, pathway, proliferation

## Abstract

**Background:**

To investigate the expression and biological functions of mitogen‐induced gene 6 (Mig‐6) in esophageal squamous cell carcinoma (ESCC).

**Methods:**

The expression of Mig‐6 in ESCC tissues and normal esophageal epithelial tissues were measured by immunohistochemistry (IHC) assay. MTT test was applied to detect the proliferative ability of ESCC cells after Mig‐6 was upregulated by transfection. A fluid cytology assay was used to detect apoptosis of ESCC cells. Agilent whole human genome oligo microarray was used to screen different expressed genes and the possible signaling pathways which might be involved.

**Results:**

The expression of Mig‐6 protein was lower in ESCC tissues compared to normal esophageal epithelial tissues. Mig‐6 could restrain the ESCC cell growth and induce cell apoptosis. PPAR, CAMs and MAPK signaling pathways might be involved.

**Conclusions:**

Mig‐6 might be a new tumor suppressor gene and a possible target for the specific therapy of ESCC.

## Graphical abstract

The expression of Mig‐6 protein was lower in ESCC tissues (a,b) compared to normal esophageal epithelial tissues (c,d). Mig‐6 could restrain the ESCC cell growth and induce cell apoptosis. PPAR, CAMs and MAPK signaling pathways might be involved in it. Mig‐6 might be a new Tumor Suppressor Gene and a possible target for the specific therapy of ESCC.

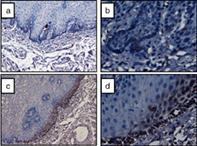

## INTRODUCTION

Esophageal cancer is one of the most common digestive system carcinomas which originates from the esophageal mucosal epithelium. The general pathological subtypes of esophageal cancer include esophageal squamous cell carcinoma (ESCC), and adenocarcinoma.^1^ Epidemiological data has demonstrated that the median incidence of esophageal cancer is 6.3 per 100 000, that it ranks seventh among all types of malignant tumors, and the mortality rate is 5.5 per 100 000, ranking sixth worldwide. A total of 572 034 new cases and 508 585 mortalities were reported in 2018. China is one of the countries with a high incidence (13.9/100 000) and mortality (12.7/100 000) of ESCC globally. It has been estimated that new cases and deaths in China account for 53.7% and 55.7%, respectively of all the ESCC cases globally.^2^ The predominant pathological subtype (more than 95%) of esophageal cancer in China is ESCC rather than adenocarcinoma. Due to the inconspicuous clinical symptoms of early esophageal cancer, most cases are at an advanced stage when diagnosed. The general prognosis is poor even for cases with early‐stage esophageal cancer undergoing surgical intervention, and local recurrence and distant metastasis are common.^3^, ^4^, ^5^ Therefore, the treatment effect of esophageal cancer is far from satisfactory, with a 5‐year overall survival rate of only 30%–40%.^6^ Therefore searching for sensitive and specific postoperative monitoring biological markers and the development of relevant targeted therapies have become research hotspots in recent years.

Mitogen‐induced gene 6 (Mig‐6) was first isolated and identified in a steroid‐induced rat liver cDNA. Mig‐6 is considered as an immediate early response gene which can be rapidly and robustly induced by stimuli including hormones, growth factors, and stresses, etc.^7^, ^8^

Mig‐6 encodes a nonkinase scaffolding adaptor protein and plays an important role in regulating stress response, maintaining homeostasis, and functioning as a tumor suppressor in multiple kinds of human cancers.^9^It has previously been reported that decreased expression of Mig‐6 was observed in multiple human cancers including breast carcinoma, non small‐ ell lung cancer, hepatocellular carcinoma, endometrial disease, papillary thyroid cancer,glioma, etc.^10^, ^11^, ^12^, ^13^, ^14^, ^15^

As a tumor suppressor, Mig‐6 can inhibit proliferation, invasion and metastasis of tumor cells and induce apoptosis. However, studies about Mig‐6 in the occurrence and development of ESCC are rare, and the relevant mechanisms remain unclear. In the present study, we aimed to clarify whether Mig‐6 protein expression is suppressed in ESCC, to investigate the effects of Mig‐6 and explore the potential signaling pathways that might be involved.

## METHODS

### Patients

Ninety paired formalin‐fixed paraffin‐embedded tissue samples (ESCC tissues and normal esophageal epithelial tissues 5 cm away from the tumor) were obtained from patients who were admitted to the Department of Cardiothoracic Surgery Tianjin Medical University General Hospital during 2019 and 2020. Written informed consent was obtained from all the cases included in the study. The Inclusion criteria were as follows: (1) Clinical stage between I to III based on pathological diagnosis without distant metastasis and surgical contraindications, (2) no preoperative chemotherapy radiotherapy or immunotherapy and (3) complete R0 resection of the tumor and two or three field lymph node dissection.

### Immunohistochemistry (IHC) staining and assessment

Conventional streptomycin‐horseradish peroxidase (HRP) method was applied in the evaluation of Mig‐6 expression in ESCC and normal esophageal epithelial tissues. ESCC and normal esophageal epithelial tissues were diluted at the volume ratio of 1:500 with the primary Mig‐6 antibody, labeled with an HRP conjugated secondary antibody (Zhongshan Jinqiao Biotechnology) and stained with 3,3‐diaminobenzidine. Two independent pathologists who were not informed of any clinical data completed the evaluation.

### Cell culture and cell transfection

Three human ESCC cell lines TE13, EC109, and KYSE510 were purchased from ATCC, incubated in RPMI1640 medium (Gibco‐BRL), supplemented with 10% fetal bovine serum (FBS) (Gibco‐BRL), 100 U/ml penicillin and 100 mg/ml streptomycin (Life Technologies), and maintained at 37°C in a humidified incubator with 5% CO_2_ atmosphere.

Mig‐6 expression vector was constructed by subcloning the full‐length coding sequence into pcDNA3.1 plasmid. The empty pcDNA‐3.1 plasmid was used as a negative control. All transfections were conducted using Lipofectamine 2000 (Invitrogen) according to the manufacturer's protocols. The mRNA and protein levels were assessed 48 h following transfection.

### RNA extraction and quantitative real‐time PCR (qRT‐PCR) assay

Total RNA was isolated from ESCC cells with Trizol reagent (Invitrogen) according to the manufacturer's protocols. NanoDrop 2000 (Thermo Scientific) was used to detect the purity and quality. Reverse transcribed 2 μg RNA using reverse transcriptase M‐MLV (Takara Bio Inc.), ribonuclease inhibitor (Takara Bio Inc.) and dNTP mixture (Takara Bio Inc.). The RT products were 1:5 diluted and subjected to quantitative polymerase chain reaction (PCR) using a SYBR Green PCR Kit (Takara Bio Inc.) on a Roche 480 Real‐Time PCR System (Roche). GAPDH was applied for the endogenous reference gene. The expression level of Mig‐6 mRNA was presented as 2^‐averageΔΔCT^ × 100%. The primers sequences used are listed in Table 1. The experiment was performed in triplicate.

**TABLE 1:** Primer sequences for qRT‐PCR

Primer	Sequence
Mig‐6	
Forward	5′‐TCTTCCACCGTTGCCAATCT‐3′
Reverse	5′‐TTCGCCTGCCAGGAACATC‐3′
GAPDH	
Forward	5′‐GAAGGTGAAGGTCGGAGTC‐3′
Reverse	5′‐GGGTGGAATCATATTGGAAC‐3′

### Protein extraction and Western blot

Total proteins were extracted from ESCC cells by cell lysis buffer and quantified by BCA protein assay kit (Beyotime Biosciences). The protein samples were then separated by 8% sodium dodecyl sulfate polyacrylamide gel electrophoresis (SDS‐PAGE). After transferring the polyvinylidene fluoride (PVDF) membranes (Millipore) were incubated with the Mig‐6 and β‐actin (Cell Signaling Technology) antibodies at 4°C overnight. Protein expression was visualized by ECL (Thermo Fisher Scientific) and detected by Chemidoc MP Imaging System (Bio‐Rad).

### MTT assay

TE13 cells were seeded in 96‐wells plates, and after 24 h the experimental group was transfected with eukaryotic expression vectors pcDNA3.1(+)/Mig‐6 and the comparison group was transfected with pcDNA3.1(+) using Lipofectamine 2000. After 24 h, one of the 96‐well plates was taken for the next examination: 20 μl MTT solution (5 mg/ml) was added, and the upper clear part was removed. A495 was measured by spectrophotometer. Restraining rate of cell proliferation = (comparison group A495‐experiment group A495)/comparison group A495. The same steps of examination were repeated after 48 and 72 h. The examinations were repeated three times.

### Flow cytometry

Two groups of ESCC cells TE13 were harvested after transfection, washed twice with cold PBS and then fixed in 70% ethanol overnight. The fixed cells were stained with propidium iodide and RNaseA for 3 h at 37°C in the dark. FACS flow cytometry was used to detect cell apoptosis. The experiment was repeated three times. Results were analyzed using ModFit software.

### Gene microarray

Two groups of ESCC cells TE13 were harvested after transfection. Agilent whole human genome oligo microarray was used to determine the changes in gene expression. The data was analyzed using the SBC Analysis System.

### Statistical analysis

Statistical analysis was performed using SPSS 23.0 statistical software (http://www-01.ibm.com/software/analytics/spss/). The measurement data is presented as mean ± standard deviation (SD). The results of real‐time PCR were analyzed by comparison of 2^‐averageΔΔCT^ × 100%. Student0027s *t*‐test or ANOVA were used to analyze corresponding data. Two tailed *p*‐values <0.05 were considered statistically significant.

## RESULTS

### Mig‐6 expression was suppressed in ESCC

**FIGURE 1:**
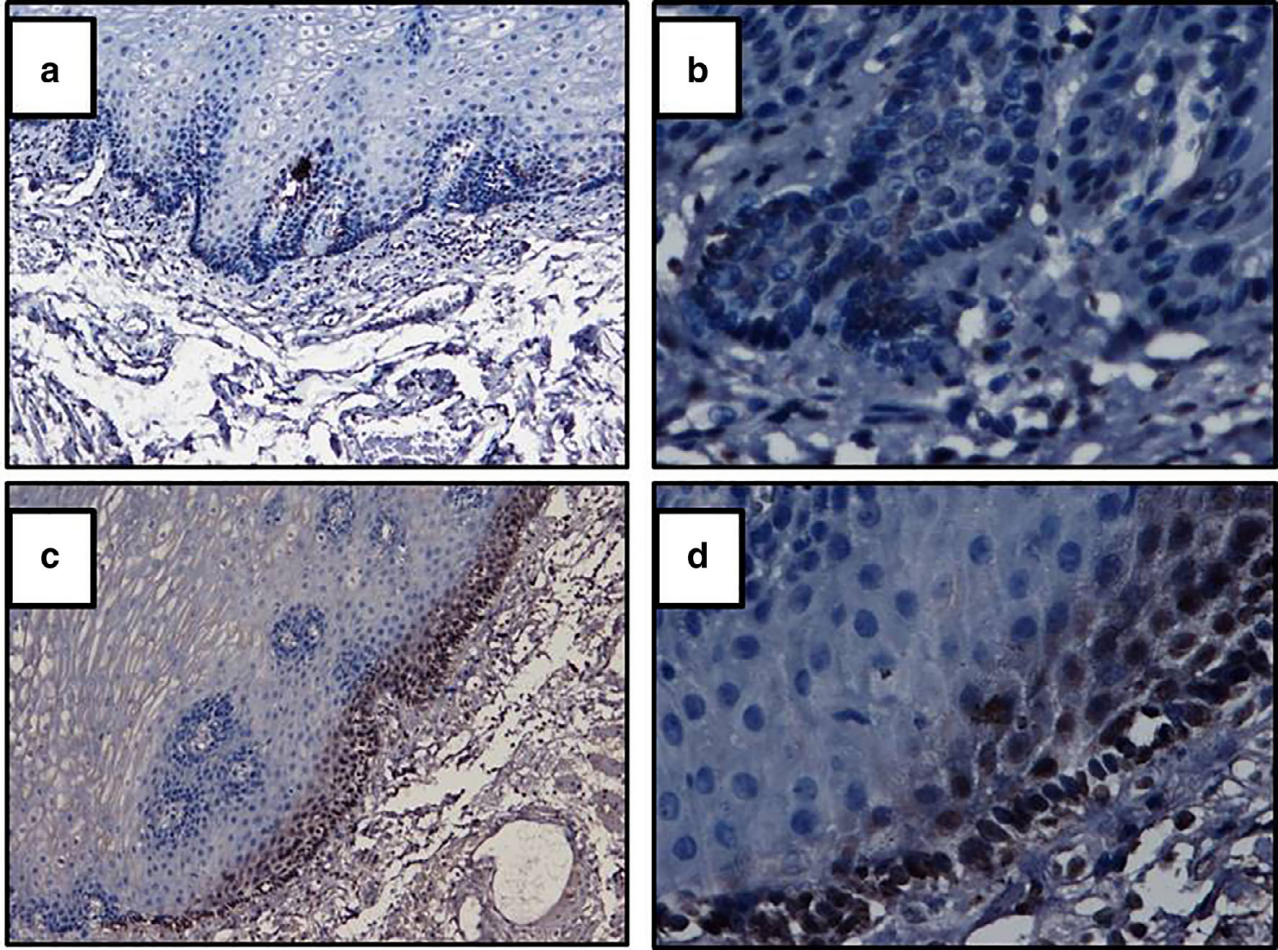
Mig‐6 protein expression in human ESCC tissues and normal esophageal epithelial tissues,((a,b) ESCC tissues, (c,d) normal esophageal epithelial tissues, (a,c) SP × 100, (b,d) SP × 400)

Immunohistochemistry analysis shows that in ESCC tissues the expression of Mig‐6 protein was significantly lower than corresponding normal esophageal epithelial tissues (Figure 1). In human ESCC tissues the positive expression of Mig‐6 protein was 26.7% (24/90), while the positive expression of Mig‐6 protein in corresponding normal esophageal epithelial tissues was 61.1% (55/90), with statistical difference (χ^2^ = 21.679,*p* < 0.05).

### Mig‐6 mRNA was lower in ESCC cell line TE13

**FIGURE 2:**
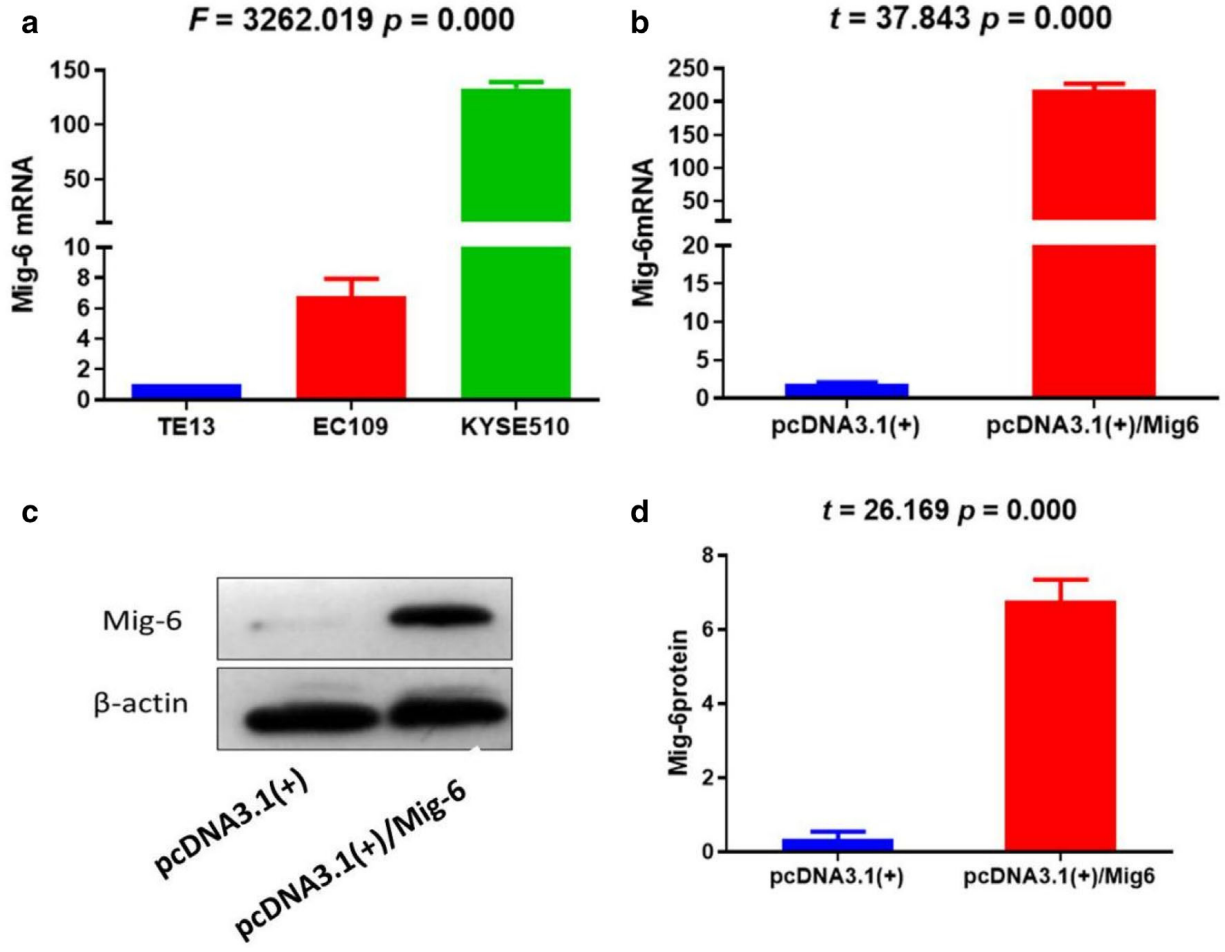
Expression of Mig‐6 mRNA in ESCC cells ((a) Mig‐6 gene mRNA expression in ESCC cell lines TE13, EC109 and KYSE510; (b) The expression of Mig‐6 mRNA after transfection by pcDNA3.1(+) and pcDNA3.1(+)/Mig‐6; (c,d) Expression of Mig‐6 protein in TE13 after transfection by pcDNA3.1(+) and pcDNA3.1(+)/Mig‐6)

Compared with EC109 and KYSE510, the relative expression level of Mig‐6 gene mRNA in TE13 was significantly lower (F = 3262.019, *p* = 0.000), which could be used as an ideal cell line for subsequent transfection experiments. To verify the efficiency of cell transfection, real‐time PCR and western blot were performed. As demonstrated in Figure 2 the expression of Mig‐6 mRNA and Mig‐6 protein in ESCC cell line TE13 were notably upregulated after transfection by pcDNA3.1(+)/Mig‐6 compared with pcDNA3.1(+).

### Mig‐6 restrained rate of ESCC cell proliferation

**FIGURE 3:**
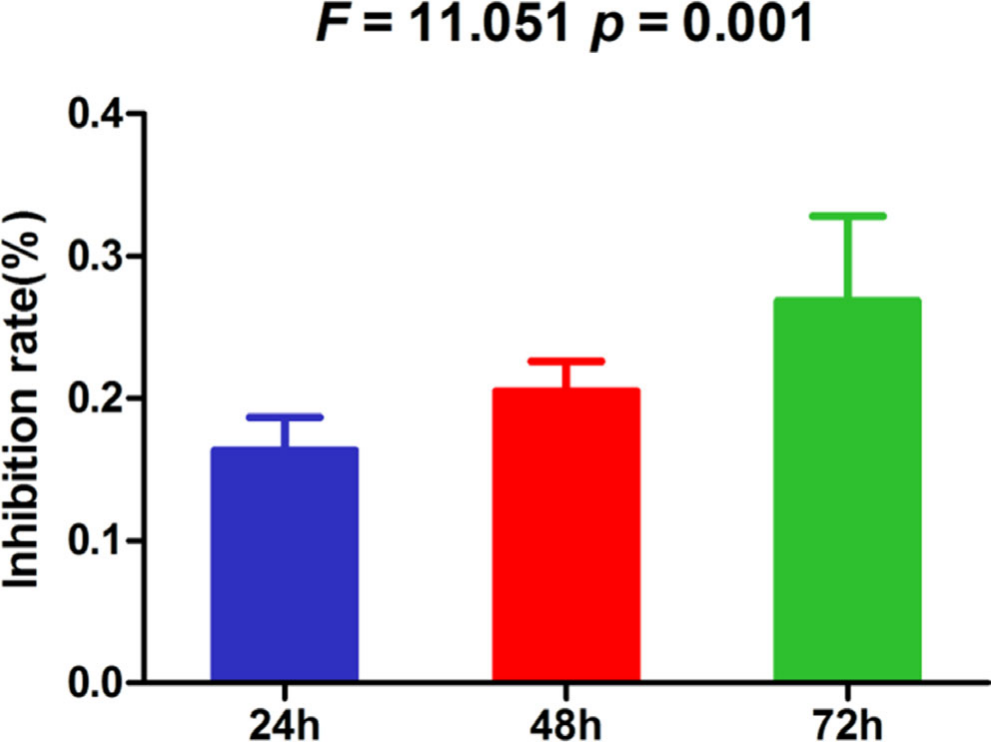
Mig‐6 inhibited the proliferation of ESCC cell TE13

MTT shows that the proliferation of ESCC cell TE13 transfected with the eukaryotic expression plasmid pcDNA3.1(+)/Mig‐6 was inhibited compared with that transfected with the empty eukaryotic expression plasmid pcDNA3.1(+). The inhibition was enhanced with the tension of time. The restraining rate of cell proliferation between the two groups was statistically significant (F = 11.051, *p* = 0.001), Figure 3.

### Mig‐6 induced apoptosis of ESCC cell

**FIGURE 4:**
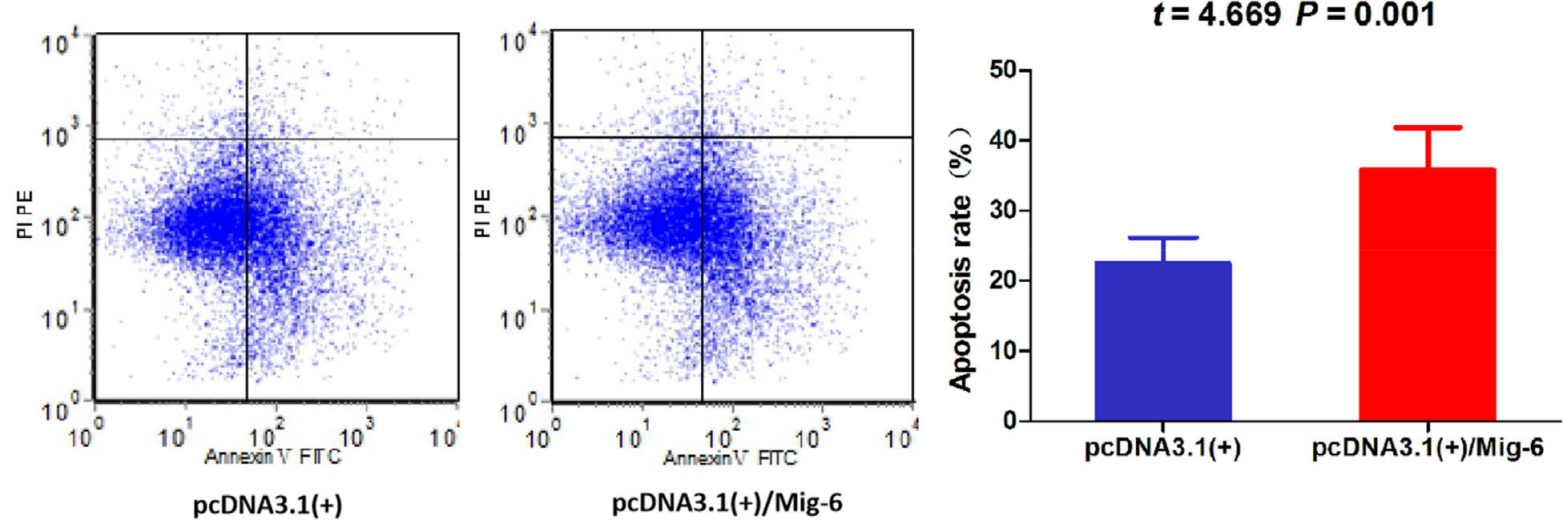
Mig‐6 increased the apoptosis of ESCC cell TE13

Flow cytometry results showed that the apoptosis of ESCC cell TE13 transfected with the eukaryotic expression plasmid pcDNA3.1(+)/Mig‐6 was increased compared with that transfected with the empty eukaryotic expression plasmid pcDNA3.1(+). The difference between the two groups was statistically significant (t = 4.669, *p* = 0.001), Figure 4.

### Mig‐6 might function as a tumor suppressor gene in ESCC through PPAR, CAMs and MAPK signaling pathways

**FIGURE 5:**
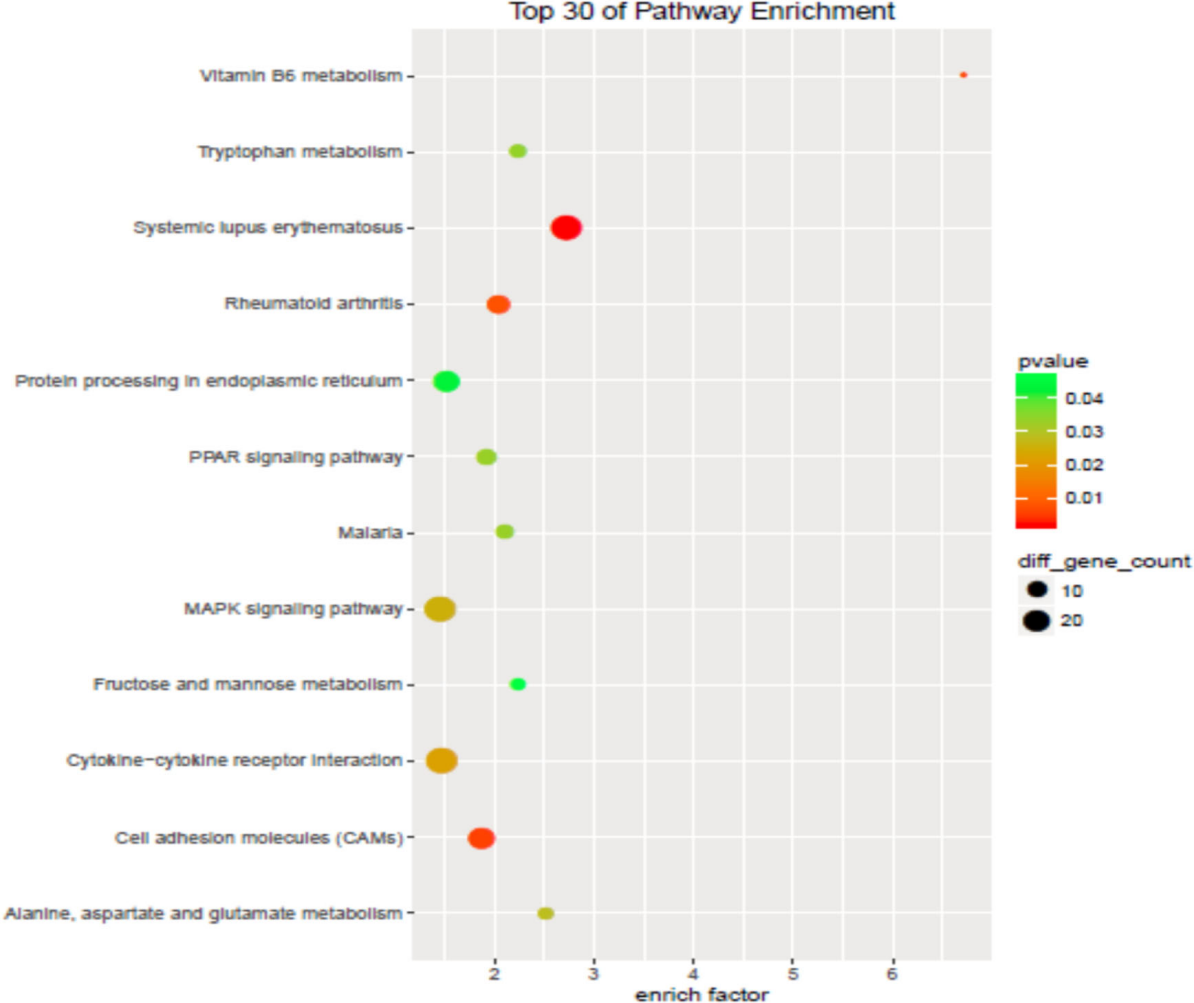
KEGG/pathway enrichment analysis

After transfection by pcDNA3.1(+)/Mig‐6 compared with pcDNA3.1(+) in ESCC cell line TE13, 1141 genes were upregulated and 1071 downregulated. KEGG pathway enrichment analysis showed that the different genes were mainly enrichment in PPAR, CAMs, and MAPK signaling pathways (Figure 5). Mig‐6 might therefore function as an suppressor gene in ESCC through these pathways.

## DISCUSSION

Mitogen‐induced gene 6 (Mig‐6), also known as ERRFi1 (ErbB receptor feedback inhibitor 1), RALT (receptor associated late transducer) and GENE33,^16^ is located on chromosome 1p36. Human chromosome 1p36 is a region rich in tumor suppressor genes, and heterozygosity deletion in this region has been observed in a variety of human malignant tumors including lung, breast, stomach, colorectal, endometrial, and ovarian cancers, sarcoma, etc.^17^ In our previous study we explored the region 1p36 in ESCC and found a new tumor suppressor gene retinoblastoma protein which binds to the zinc finger gene 1 (RIZ1). We found that the expression levels of RIZ1 mRNA and protein in ESCC tissues were lower than those in normal esophageal epithelial tissues. The deactivation of RIZ1 was associated with highly methylation in DNA promoter region and could induce imbalance expression of RIZ1/RIZ2. The imbalance expression of RIZ1/RIZ2 contributes to the progression of ESCC.^18^, ^19^ The occurrence and development of ESCC is a complex process involving multiple factors, multiple genes changing and multiple stages of carcinogenesis. Mig‐6 located in this region has tumor‐inhibiting activity in a variety of malignant human cancers. However, there are currently few studies on Mig‐6 in ESCC.

In this study, we first detected the expression of Mig‐6 protein in ESCC tissues and normal esophageal epithelial tissues by immunohistochemical assay. The results showed that Mig‐6 protein was mainly expressed in cytoplasm. The positive rate of Mig‐6 protein expression in ESCC tissues was significantly lower than that in normal esophageal epithelial tissues. In order to clarify the biological functions of Mig‐6 in ESCC, TE13 cell line with low expression of Mig‐6 gene mRNA was selected as the experimental cell line. Mig‐6 expression was upregulated by transfection. MTT assay results showed that the inhibition rate of cell growth increased with the prolongation of time. Mig‐6 had a significant inhibitory effect on the proliferation of ESCC cells. Flow cytometry showed that upregulation of Mig‐6 promoted apoptosis of ESCC cells. In vitro experiments confirmed the anticancer activity of Mig‐6 in ESCC. This study might therefore provide a theoretical and experimental basis for gene targeted precision therapy of ESCC.

Mig‐6 protein consists of several domains including Cdc42/Rac‐interaction and binding (CRIB) domain, Src‐homology (SH3) binding moieties, 14‐3‐3 protein‐binding motif and an Ack1 homology (AH) domain that contains EGFR binding segment.^20^

Mig‐6 can directly interact with all four members of the ErbB family, including epidermal growth factor receptor (EGFR) and ErbB2‐4. It acts as a negative feedback regulator of EGFR signaling in human malignant tumors.^21^ In this study, gene microarray results showed that Mig‐6 might function as an tumor suppressor gene in ESCC through PPAR, CAMs, and MAPK signaling pathways, etc. The specific mechanism for this requires further extensive studies. Further follow‐up data and statistics are needed to determine whether Mig‐6 play a role in the prognostic assessment and recurrence monitoring of ESCC. We hope to carry out these studies in the future.

## CONFLICT OF INTEREST

The authors do not report any conflict of interest.
